# Persuasiveness of Statistics and Patients’ and Mothers’ Narratives in Human Papillomavirus Vaccine Recommendation Messages: A Randomized Controlled Study in Japan

**DOI:** 10.3389/fpubh.2018.00105

**Published:** 2018-04-12

**Authors:** Tsuyoshi Okuhara, Hirono Ishikawa, Masafumi Okada, Mio Kato, Takahiro Kiuchi

**Affiliations:** Department of Health Communication, School of Public Health, The University of Tokyo, Tokyo, Japan

**Keywords:** human papillomavirus vaccination, anti-vaccination movement, narrative, persuasion, health communication

## Abstract

**Background:**

The human papillomavirus (HPV) vaccination percentage among age-eligible girls in Japan is only in the single digits. This signals the need for effective vaccine communication tactics. This study aimed to examine the influence of statistical data and narrative HPV vaccination recommendation massages on recipients’ vaccination intentions.

**Methods:**

This randomized controlled study covered 1,432 mothers who had daughters aged 12–16 years. It compared message persuasiveness among four conditions: statistical messages only; narrative messages of a patient who experienced cervical cancer, in addition to statistical messages; narrative messages of a mother whose daughter experienced cervical cancer, in addition to statistical messages; and a control. Vaccination intentions to have one’s daughter(s) receive the HPV vaccine before and after reading intervention materials were assessed. Statistical analysis was conducted using analysis of variance with Tukey’s test or Games–Howell *post hoc* test, and analysis of covariance with Bonferroni correction.

**Results:**

Vaccination intentions after intervention in the three intervention conditions were higher than the control condition (*p* < 0.001). A mother’s narrative messages in addition to statistical messages increased HPV vaccination intention the most of all tested intervention conditions. A significant difference in the estimated means of intention with the covariate adjustment for baseline value (i.e., intention before intervention) was found between a mother’s narrative messages in addition to statistical messages and statistical messages only (*p* = 0.040).

**Discussion:**

Mothers’ narrative messages may be persuasive when targeting mothers for promoting HPV vaccination. This may be because mothers can easily relate to and identify with communications from other mothers. However, for effective HPV vaccine communication, further studies are needed to understand more about persuasive differences in terms of statistics, narratives, and narrators. Directions for future research are also suggested.

## Introduction

In communicating about risks and benefits of public health practices, it is essential to convey evidence-based information and to influence the audience to make better health decisions (1). This concept of public health communication is especially important in the current human papillomavirus (HPV) vaccination crisis in Japan. The HPV vaccination rate for age-eligible girls in Japan was as high as 70–80% in 2011 and 2012 (2). However, owing to a series of negative campaigns by mass media on severe adverse reactions allegedly caused by HPV vaccination, such as difficulties walking and memory impairment, the proactive recommendation of HPV vaccination was suspended by the Ministry of Health, Labor, and Welfare in June 2013. As a direct result, the HPV vaccination rate among age-eligible girls fell sharply to only a few percent by 2014 (2). Fears concerning adverse reactions to HPV vaccination are now a significant reason for avoiding vaccination in Japan and other countries (3–7), despite studies demonstrating the safety of HPV vaccines (8, 9). In this critical situation, conveying scientific information alone may not sway the biased anti-HPV vaccination sentiment; influential and persuasive communication tactics to encourage the audience to make less biased decisions are needed.

In Japan, unlike in many other developed countries, the primary care doctor system is underdeveloped, and school-administrated HPV vaccination has not been routinely performed. Most mothers and their daughters do not have health professionals whom they can consult with about HPV vaccines. They gather information independently and judge by themselves whether or not vaccination will be sought. Accordingly, when proactive recommendation of HPV vaccination resumes in the future, publicizing online and offline vaccination recommendation messages from the Ministry of Health, Labor, and Welfare and municipalities will be a means for recommending vaccination to mothers and their daughters. Therefore, examining persuasive messages that recommend HPV vaccination is an urgent issue in Japan.

Since Aristotle, the use of evidence has been a primary means of enhancing message persuasiveness. Evidence is the set of factual statements that originate from a source other than the communicator and are offered to verify the communicator’s claims (10). Evidence can be divided broadly into two categories: statistical and narrative evidence (11). Statistical evidence, such as frequencies and percentages, provide proof in the form of summary information across a larger number of cases (11); e.g., “The frequency of severe adverse reactions to the HPV vaccine, such as acute disseminated encephalomyelitis, is one in 4.3 million.” Narrative evidence refers to the use of case stories or examples to support the argument offered by the communicator (11); e.g., “I am suffering from the aftereffect of cervical cancer. Therefore, I recommend you receive the HPV vaccine to prevent cervical cancer.” Studies indicate that presenting statistical or narrative evidence almost always enhances message persuasiveness (10–12).

A primary question among scholars has focused on the relative persuasiveness of messages presenting statistical evidence compared with those presenting narrative evidence. At present, study results are somewhat contradictory, and no definitive difference has been demonstrated between narrative and statistical evidence (10–14). However, using narratives to motivate health behavior is an emerging form of persuasion in public health (15–17). Several recent studies in the context of vaccine communication show that narrative messages about experiences of disease increase the audience’s risk perception of developing the disease, vaccination intention, and behaviors to prevent the disease to a greater degree than do didactic messages (18–21). Accordingly, use of narrative messages in addition to evidence-based statistical messages to counter the influence of anti-vaccination narratives of alleged victims of vaccine adverse reactions has been proposed (22–25). Thus, our first research question emerges herein: *will a narrative message in addition to statistical messages result in higher intention to receive the HPV vaccine than statistical messages only?*

Several models have been proposed to explain the persuasiveness of narratives, including the transportation-imagery model (26), extended elaboration likelihood model (27), and entertainment overcoming resistance model (28). These models share the audience’s identification with a character of a narrative and transportation (i.e., caught up in, or carried away by, the story) as factors that serve to enhance the persuasiveness of narratives. Among various factors that have been explored, identification and transportation are the primary factors presently warranted by the literature (29).

Although the relationship of the influence of identification and transportation on narrative persuasion is not entirely clear, identification may foster transportation and consequently enhance persuasiveness (30, 31). Identification is a mechanism through which audience members experience reception and interpretation of the text internally, as if the events were happening to them (32). During identification, the audience imagines that he or she becomes the character and replaces his or her personal identity and role as an audience member with the identity and role of the character (32). A number of studies show that greater identification with characters is associated with greater persuasiveness of narratives (33–40). A meta-analysis reported that perceived identification produced statistically significant effects on forming of story-consistent attitudes, beliefs, and behaviors on recipients (41). In addition, in the context of HPV vaccine communication, a study showed that the first-person narrative of a college student resulted in higher perceived risk of HPV infection than a report in the third-person narrative among participants who were college students (20). The authors discussed that the first-person narrative of a peer was easier to identify with for participants (20). Another study of college students found more HPV vaccine inoculation behaviors in the group who viewed the narrative of a college student than the narrative of a medical expert (21). Similarly, it was thought that participants could more easily identify with the narrative of a peer.

Japanese refer to the HPV vaccine as “sikyu keigan wakuchin” (i.e., cervical cancer vaccine). Therefore, a narrative of a cervical cancer patient who recommends HPV vaccination may be persuasive for some audiences. However, healthy individuals may have difficulty identifying with a patient. When targeting mothers to promote HPV vaccination, the narrative of a mother who has a daughter may be easier to identify with and more persuasive than the narrative of a patient. Thus, our second research question emerges herein: *will the narrative of a mother whose daughter experienced cervical cancer result in higher intention to have their daughters receive the HPV vaccine than the narrative of a patient who experienced cervical cancer among participants who are mothers with daughters?*

This study aimed to examine the influence of HPV vaccination recommendation messages. It did so by using statistical data and narratives on vaccination intention of mothers with daughters aged 12–16 years, in Japan. This will help ensure that persuasive vaccine recommendation messages will be disseminated when proactive recommendation of the vaccination eventually resumes. A randomized controlled study was conducted to answer the above two research questions.

## Materials and Methods

### Study Design and Participants

Participants were recruited from persons registered in a survey company database in Japan. Eligibility criteria were mothers who have a daughter(s) aged 12–16 years who has never received HPV vaccination. An e-mail was sent to registered persons, which described that questionnaires (and materials) were created and intended for research purposes only. Recipients who were eligible and consented to participate in the study were invited to a web-based survey. After conducting a pretest among 120 participants in August 2017, a total of 1,432 mothers excluding pretest participants completed the survey in September 2017.

When participants consented to participate in the study on the web screen, they were randomly assigned to a group that received statistical messages only, a group that received a patient’s narrative messages in addition to statistical messages, a group that received a mother’s narrative messages in addition to statistical messages, or a control group by algorithm included in the web-based survey computer program. Because required sample size in each intervention group was 394 participants (this will be discussed later), recruiting stopped when the number of participants of all of the intervention groups reached 394. All participants were asked about items such as sociodemographic information, history of cancer, and sexually transmitted disease, and whether they knew about the media coverage of adverse reactions to the HPV vaccine. Participants in the intervention groups were asked their intention to have their daughter(s) receive the HPV vaccine before and after reading the intervention material. They were also asked their attitude toward HPV vaccination after reading the material. Participants in the control group were asked their intention of vaccination without reading intervention materials. Token gifts were given to all participants upon completion of the study by the survey company. The protocol was approved by the ethical review committee at the Graduate School of Medicine, The University of Tokyo. All subjects gave written informed consent in accordance with the Declaration of Helsinki.

### Intervention Materials

Statistical content on the materials of the three intervention groups was taken from the websites of the Ministry of Health, Labor, and Welfare; National Cancer Center Japan; and a consensus statement from 17 relevant Japanese academic societies on the promotion of the HPV vaccine (9). The statistical content included cervical cancer morbidity and mortality and HPV vaccine efficacy and safety. The content was identical among the three intervention materials. The statistical message contained a total of 745 Japanese characters.

Narrative contents of a patient and a mother were taken from a website of the United States Centers for Disease Control and Prevention (42) and modified for this study. In the narrative content, the narrator told the experience of being diagnosed with cervical cancer, having a total hysterectomy, giving up the dream of having children, suffering from complications, fearing cancer recurrence, and recommending HPV vaccination. These narrative contents were identical between the two intervention materials (patient’s and mother’s narratives) except for the subject of the narrative (i.e., “I,” in the patient’s narrative and “my daughter” in the mother’s narrative). The patient’s and mother’s narratives contained a total number of 341 and 357 Japanese characters, respectively. Appendix S1 in Supplementary Material shows the statistical messages and mother’s narrative used in this study, which was translated into English for this report.

### Outcome Measures

The primary outcome was intention to have one’s daughter(s) receive the HPV vaccine after intervention. Intention before intervention was assessed for covariate adjustment for baseline value and changes in intention before and after intervention. Participants responded to the following three questions on 1–6 scales ranged from “extremely unlikely,” “unlikely,” “a little unlikely,” “a little likely,” “likely,” to “extremely likely”: (1) “How likely would you have your daughter(s) receive the HPV vaccine sometime soon?”; (2) “If you were faced with the decision of whether to have your daughter(s) receive the HPV vaccine today, how likely is it that you would choose to have her receive the vaccine?”; and (3) “How likely would you have your daughter(s) receive the HPV vaccine in the future?” A summary score calculated by dividing the sum of scores of the three questions by three (i.e., mean) was used in the analysis. Higher scores indicate greater intentions. This measure was adapted from a previous study (43). The secondary outcome was attitude toward HPV vaccination. Participants rated “having my daughter(s) receive the HPV vaccine” on a scale consisting of five 1–6 semantic differential items (bad/good, harmful/beneficial, foolish/wise, threatening/assuring, and risky/safe). A summary score calculated by dividing the sum of scores of the five items by five (i.e., mean) was used in the analysis. Higher scores indicate more favorable attitudes. This measure was adapted from a previous study (44).

### Sample Size

A previous study of HPV vaccination communication showed that the effect sizes for comparing vaccination intention between “statistics only” and “a narrative in addition to statistics” conditions and between a peer’s and a third-person’s narrative conditions were small (Cohen’s *d* = 0.2) (20). Based on these results, we conducted a power analysis using the G*Power 3 program (45, 46) to determine sample size. This analysis showed that 394 participants were required in each of the three intervention groups to detect an effect size (Cohen’s *d*) of 0.2 for comparing vaccination intention between “statistics only” and “a narrative in addition to statistics” conditions and between patient’s and mother’s narrative conditions at an alpha error rate of 0.05 (two-tailed) and a beta error rate of 0.20. We set the sample size at about 200 for the control group because the number of participants who matched the eligibility criteria was limited among persons registered with the survey company.

### Statistical Analysis

Descriptive statistics were used to describe participants’ sociodemographic information, history, and baseline intention to vaccinate, by summarizing in percentages for categorical variables and as mean ± SD for continuous variables. Analysis of variance (ANOVA) was conducted with the intention of vaccination or attitude toward vaccination as the dependent variable and the group assignment as the independent variable. Tukey’s test was conducted on significant main effects where appropriate. The Games–Howell *post hoc* test was performed when the assumption of homogeneity of variances was not satisfied. According to recommendations in the literature including the Consolidated Standards of Reporting Trials statement, significance testing of baseline differences and covariate adjustment for variables that differ at baseline are not recommended because such adjustment is likely to bias the estimated intervention effect. However, covariate adjustment for a baseline value of a quantitative outcome is recommended because a strong correlation between the baseline value and the outcome is expected (47–50). Therefore, we conducted an analysis of covariance (ANCOVA) with the baseline intention (i.e., before reading the intervention materials) as the covariate. Bonferroni correction was applied for the *post hoc* test to compare adjusted means of intentions. In addition, intention of vaccination was compared between before and after intervention using the paired *t*-test. ANOVA was conducted with changes in intention of vaccination before and after intervention as the dependent variable and the group assignment as the independent variable. Tukey’s test was conducted on significant main effects where appropriate. A *p*-value of <0.05 was set as significant in all statistical tests. All following *p*-values presented in the results of Tukey’s test, the Games–Howell *post hoc* test, and Bonferroni *post hoc* analyses were adjusted for multiple comparison. All statistical analyses were performed using IBM SPSS Statistics for Windows, Version 21.0 (IBM Corp., Armonk, NY, USA).

## Results

### Participant Characteristics

Numbers of participants were 394 in a group that received statistical messages only, 408 in a group that received a patient’s narrative messages in addition to statistical messages, 411 in a group that received a mother’s narrative messages in addition to statistical messages, and 219 in a control group. Table 1 shows the participants’ baseline characteristics. Participant age ranged from 30 to 61 years (mean = 44 years, SD = 4.7). 49.7% of their daughters were 12–14 years old, and the remaining were 15–16 years old. Participants were distributed throughout Japan. About 90% of participants were not advised by health professionals to have their daughter(s) receive the HPV vaccine. About 90% of participants knew the media coverage of adverse reactions to the HPV vaccine and suspension of the proactive recommendation for HPV vaccination by the government. About 10% of participants had histories of cervical cancer or sexually transmitted disease personally or familiar persons.

**Table 1:** Participant sociodemographic information, history, and baseline intention of vaccination.

	Statistics only (*n* = 394)	Statistics and patient’s narrative (*n* = 408)	Statistics and mother’s narrative (*n* = 411)	Control (*n* = 219)	Total (*n* = 1,432)
Age, mean years (SD)	44.2 (4.6)	43.9 (4.6)	44.2 (4.9)	43.8 (4.6)	44.1 (4.7)
Age of daughters, %					
12–14 years old	50.5	49.0	48.9	50.7	49.7
15–16 years old	49.5	51.0	51.1	49.3	50.3
Highest education, %					
Less than high school	3.3	2.7	3.4	3.2	3.1
High school graduate	30.2	30.6	29.2	31.5	30.2
Some college	41.1	40.4	43.8	47.5	42.7
College graduate	23.6	25.5	23.1	17.4	23.1
Graduate school	1.8	0.7	0.5	0.5	0.9
Household income, %					
Less than 2 million yen^a^	7.4	7.1	7.1	10.5	7.7
2–6 million yen^a^	34.5	36.5	37.0	34.2	35.8
More than 6 million yen^a^	43.4	42.9	41.6	42.5	42.6
Unknown	14.7	13.5	14.4	12.8	14.0
Advised by health professionals to have their daughter(s) receive human papillomavirus (HPV) vaccines, %					
Yes	8.1	6.1	6.3	5.5	6.6
No	91.9	93.9	93.7	94.5	93.4
Knew about media coverage of adverse reactions to HPV vaccines, %					
Yes	87.6	89.2	91.5	93.6	90.1
No	12.4	10.8	8.5	6.4	9.9
Knew about suspension of the proactive recommendation for HPV vaccination by the government, %					
Yes	84.0	86.5	86.9	86.3	85.9
No	16.0	13.5	13.1	13.7	14.1
History of cervical cancer including familiar persons, %					
Yes	7.4	10.0	9.5	8.2	8.9
No	92.4	89.0	90.5	91.3	90.7
No answer	0.3	1.0	0	0.5	0.4
History of cancer other than cervical cancer including familiar persons, %					
Yes	15.7	21.3	17.5	19.2	18.4
No	84.0	78.2	82.0	80.8	81.3
No answer	0.3	0.5	0.5	0	0.3
History of sexually transmitted disease including familiar persons, %					
Yes	7.6	9.8	8.3	7.3	8.4
No	92.1	89.2	90.0	91.8	90.6
No answer	0.3	1.0	1.7	0.9	1.0
Intention of vaccination before reading the intervention material, mean (SD)	2.56 (0.96)	2.53 (0.91)	2.41 (0.94)	–	2.50 (0.94)

*^a^One US dollar is roughly equivalent to 100 yen*.

### Intervention Effect

Internal consistencies of questions about intention to have one’s daughter(s) receive the HPV vaccine and questions about attitude toward vaccination were excellent (Cronbach’s α = 0.952, M = 2.73, SD = 0.99 in intention; Cronbach’s α = 0.945, M = 3.02, SD = 0.98 in attitude). Table 2 shows the intention of and attitude toward HPV vaccination across groups. Regarding intention of vaccination, ANOVA revealed a main effect of the group assignment [*F*(3, 1,428) = 17.198, MSE = 0.957, *p* < 0.001, η^2^ = 0.035]. Tukey’s test revealed a significant difference between the control and the statistics only (M = 2.30 vs. M = 2.81, *p* < 0.001), the control and the patient’s narrative in addition to statistics (M = 2.30 vs. M = 2.85, *p* < 0.001), and the control and the mother’s narrative in addition to statistics (M = 2.30 vs. M = 2.77, *p* < 0.001) groups. There was no significant difference in intention of vaccination between the three intervention groups. Regarding attitude toward vaccination, ANOVA revealed a main effect of the group assignment [*F*(3, 1,428) = 42.553, MSE = 1.645, *p* < 0.001, η^2^ = 0.082]. The Games–Howell *post hoc* test revealed a significant difference between the control and the statistics only (M = 2.39 vs. M = 3.10, *p* < 0.001), the control and the patient’s narrative in addition to statistics (M = 2.39 vs. M = 3.21, *p* < 0.001), and the control and the mother’s narrative in addition to statistics (M = 2.39 vs. M = 3.12, *p* < 0.001) groups. There was no significant difference in attitude between the three intervention groups.

**Table 2:** Intention of and attitude toward human papillomavirus vaccination across groups.

	Statistics only (*n* = 394)	Patient’s narrative in addition to statistics (*n* = 408)	Mother’s narrative in addition to statistics (*n* = 411)	Control (*n* = 219)	*p*
Intention of vaccination, mean (SD)^a^	2.81 (1.00)	2.85 (1.01)	2.77 (0.96)	2.30 (0.90)	<0.001
Attitude toward vaccination, mean (SD)	3.10 (1.00)	3.21 (0.98)	3.12 (0.90)	2.39 (0.85)	<0.001
Intention of vaccination, estimated mean after adjusting for baseline intention (95% CI)^a^	2.76 (2.71–2.81)	2.82 (2.77–2.87)	2.84 (2.80–2.89)	–	0.038

*CI, confidence interval*.

*^a^Intention after intervention except for a control condition*.

Analysis of covariance with the baseline intention set as the covariate revealed the main effect of the group assignment [*F*(2, 1,209) = 3.287, MSE = 0.255, *p* = 0.038, η^2^ = 0.0014]. The estimated means of intention after intervention with the covariate adjustment for baseline values are shown in Table 2. Bonferroni *post hoc* analyses revealed a significant difference between the statistics only and the mother’s narrative in addition to statistics groups (M = 2.76 vs. M = 2.84, *p* = 0.040). There was no significant difference in the estimated means of intention between the statistics only and the patient’s narrative in addition to statistics groups.

**Figure 1:**
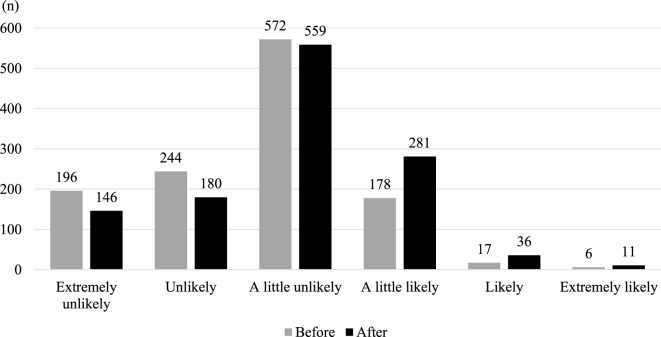
Distributions of intention of vaccination before and after intervention.

The mean of intention to have one’s daughter(s) receive the HPV vaccine before and after intervention in the three intervention groups was 2.50 (SD = 0.94) and 2.81 (SD = 0.99), respectably. Figure 1 shows distributions of intention of vaccination before and after intervention. Table 3 shows changes in intention of vaccination before and after intervention across groups. The paired *t*-test revealed significant changes in intention before and after intervention in the all intervention groups (*p* < 0.001). ANOVA revealed a main effect of the group assignment [*F*(2, 1,210) = 4.078, MSE = 0.262, *p* < 0.017, η^2^ = 0.007]. Tukey’s test revealed a significant difference of change amount between the statistics only and the mother’s narrative in addition to statistics groups (M = 0.25 vs. M = 0.35, *p* = 0.014). There was no significant difference in change amount between the statistics only and the patient’s narrative in addition to statistics groups.

**Table 3:** Changes in intention of vaccination before and after intervention across groups.

	Statistics only (*n* = 394)			Statistics and patient’s narrative (*n* = 408)			Statistics and mother’s narrative (*n* = 411)			*p*^b^
		
	Before	After	*p*^a^	Before	After	*p*^a^	Before	After	*p*^a^	
Intention of vaccination, mean (SD)	2.56 (0.96)	2.81 (1.00)	<0.001	2.53 (0.91)	2.85 (1.01)	<0.001	2.41 (0.94)	2.77 (0.96)	<0.001	

Changes in intention before and after intervention, mean (SD)	0.25 (0.49)			0.32 (0.53)			0.35 (0.52)			0.017

*^a^p-Values were assessed using the paired t-test*.

*^b^A p-value was assessed using analysis of variance*.

## Discussion

### Persuasiveness of Statistical and Narrative Evidence

We conducted a randomized controlled study to compare message persuasiveness between different conditions in terms of statistics, narratives, and narrators in the context of HPV vaccine communication. This study found that statistical only messages as well as a narrative (of a patient or a mother) in addition to statistical messages significantly improved mothers’ attitude and intention to have their daughter(s) receive the HPV vaccine than a no message condition (control). This result is consistent with a number of studies that showed presenting statistical or narrative evidence enhances message persuasiveness (10–12).

### Persuasiveness of Patient’ vs. Mother’ Narrative

One of our research questions was: will the narrative of a mother whose daughter experienced cervical cancer result in higher intention to have their daughters receive the HPV vaccine than the narrative of a patient who experienced cervical cancer among participants who are mothers with daughters? This study found no significant difference in attitude and intention of vaccination between the statistics only, patient’s narrative in addition to statistics, and mother’s narrative in addition to statistics groups. One of the reasons may be that baseline intentions affected intentions after reading the material; the baseline intention was the highest in the statistics only group (M = 2.56) and the lowest in the mother’s narrative in addition to statistics group (M = 2.41) (see Table 1). The estimated mean of intention after adjusting for baseline intentions in the mother’s narrative in addition to statistics group was the highest among intervention groups, and significantly higher than in the statistics only group. In addition, the change in intention of vaccination before and after intervention in the mother’s narrative in addition to statistics group was the largest among intervention groups, and significantly larger than in the statistics only group, although this result should be interpreted with caution because the regression to the mean of baseline values may be mixed in those changes (51). Similarity of the audience to a narrator should increase the likelihood of identification and consequently persuasiveness of the narrative message (30–41). The mother’s narrative may have been easy to feel similar to and identify with for participants who were mothers with daughters and consequently contributed to increase mothers’ vaccination intentions.

However, even after adjusting for baseline intentions, we found no significant difference in vaccination intention between the patient’s narrative in addition to statistics and the mother’s narrative in addition to statistics groups. This may be because other factors such as increase in self-efficacy through modeling (28) and generation of fewer counterarguments (26) were more important than identification with the narrator in HPV vaccine communication. Future studies focusing on other factors such as modeling may be useful; e.g., a study that uses the narrative of a mother who had her daughter receives the HPV vaccine.

### Persuasiveness of Statistics Only vs. Statistics in Addition to Narrative

Another of our research questions was: will a narrative message in addition to statistical messages result in higher intention to receive the HPV vaccine than statistical messages only under the circumstances of the HPV vaccination crisis in Japan? We found no significant difference in vaccination intention between the statistics only and the patient’s narrative in addition to statistics groups before as well as after adjusting for baseline intentions. The process of identification involves multiple dimensions such as sharing the character’s feelings and perspective, internalizing the character’s goals, and the loss of self-awareness by being absorbed in the story (32). Considering this, the narrative message used in the present study was created for research purposes and therefore may have been too short and dull without a picture or the name of the narrator to develop the process of identification for the audience. It may be useful to examine the persuasiveness of a longer and more vivid narrative of cervical cancer experience in future studies so that the audience can develop the process of identification.

### Limitations

The findings of this study must be interpreted with several limitations. First, narrative persuasion is generally hindered when the persuasive intent is obvious, as in the intervention materials of the present study, because some audiences may react against being manipulated (52). This constraint of intervention materials in this study should be noted in addition to shortness and dullness as discussed above. Second, although previous studies assessed the degree of identification with characters in terms of components such as liking, feeling like you know, and wanting to be like, we did not assess the degree of identification because such measurement items were not applicable to the narratives in the present study. However, mothers may obviously identify more with a mother’s narrative than a patient’s narrative because similarity of the audience to a narrator should increase the likelihood of identification (32). Third, this study assessed vaccination intentions directly after message exposure. The long-term effects of promotional messages should be examined in future studies because they are important in this context given that HPV vaccination requires multiple injections over a series of weeks. Fourth, this study assessed vaccination intentions rather than actual vaccination behaviors. However, measuring intention is beneficial in public health studies because it predicts actual behaviors (53). Finally, considering the critical situation of HPV vaccination in Japan, participants’ prior attitudes toward and intentions of vaccination may have been negatively distorted and affected the study results. Therefore, our results should be interpreted with caution.

## Conclusion

Statistical messages of HPV vaccine efficacy and safety, the narrative of a patient who experienced cervical cancer in addition to statistical messages, and the narrative of a mother whose daughter experienced cervical cancer in addition to statistical messages increased vaccination intention among participants who were mothers with daughters. In the practice of HPV vaccine communication, exposure to messages of HPV vaccine efficacy and safety may increase a mother’s vaccination intention whether it includes only statistical messages or narratives of cervical cancer experience in addition to statistical messages. Our study results may indicate that active dissemination of messages about efficacy and safety of HPV vaccines by public health institutions including the Ministry of Health, Labor, and Welfare and mother’s exposure to those messages are the keys to improve their attitude toward and intention of HPV vaccination.

A mother’s narrative in addition to statistical messages resulted in slightly higher vaccination intention than statistical messages only and a patient’s narrative message in addition to statistical messages. Mothers may feel similar to and easily identify with a mother’s narrative. Narrative of a mother with a daughter who experienced cervical cancer may be persuasive for audiences who are mothers. The public health institutions including the Ministry of Health, Labor, and Welfare may be able to increase persuasiveness of HPV vaccination recommendation messages by using the narrative of a mother’s daughter experienced cervical cancer in addition to statistical messages when they create materials such as leaflets and websites.

However, further studies are needed to understand more about persuasive differences between statistical and narrative messages as well as factors influencing the persuasiveness of narratives. A deeper understanding of these factors will help health professionals to more effectively communicate with individuals and communities to encourage better decision making regarding HPV vaccination.

## Ethics Statement

This study was carried out in accordance with the recommendations of the ethical review committee at the Graduate School of Medicine, The University of Tokyo. The protocol was approved by the ethical review committee at the Graduate School of Medicine, The University of Tokyo. All subjects gave written informed consent in accordance with the Declaration of Helsinki.

## Author Contributions

TO and HI conceived the concept and design of the study. TO conducted statistical analysis and wrote the manuscript. HI provided the first feedback for the manuscript. All authors discussed the manuscript critically and approved the final version.

## Conflict of Interest Statement

The authors declare that the research was conducted in the absence of any commercial or financial relationships that could be construed as a potential conflict of interest.

## Abbreviations

HPV, human papillomavirus; ANOVA, analysis of variance; ANCOVA, analysis of covariance; CONSORT, Consolidated Standards of Reporting Trials.
