# Supplementary material for: Persuasiveness of Statistics and Patients’ and Mothers’ Narratives in Human Papillomavirus Vaccine Recommendation Messages: A Randomized Controlled Study in Japan
Source: Front Public Health. 2018 Apr 12;6:105. doi: 10.3389/fpubh.2018.00105 (PMC5906532; doi:10.3389/fpubh.2018.00105)
Supplement: Supplementary file 1 [file data_sheet_1.PDF]

Appendix The intervention material used in the group that received a mother's narrative in addition to statistical messages

HPV vaccines: receive or not receive?

About cervical cancer

Cervical cancer is a disease caused by human papillomavirus (HPV) infection. HPV is transmitted by sexual intercourse and persistent HPV infection can progress to cervical cancer. Approximately 10,000 people are diagnosed with and about 3,000 people die of cervical cancer annually in Japan. Mortality due to cervical cancer has increased, and in recent years patients in their 20–30s are mainly affected.

About HPV vaccines

Girls can prevent HPV infection and cervical cancer by being vaccinated with HPV vaccines. Recommended age targets for vaccination are girls in the 6th grade of elementary school to those in the 1st grade of high school.

Efficacy of HPV vaccines

Efficacy data on HPV vaccines are now very strong.

- Many countries (65) have included HPV vaccines in their national immunization programs.
- Several of these countries (e.g., Australia, USA, Denmark, and Scotland) have reported that the incidence rate of precancerous lesions of the uterine cervix has decreased by approximately 50% since institution of widespread HPV immunization programs.

Safety of HPV vaccines

HPV vaccine safety has been confirmed in domestic and foreign surveys.

- In a domestic survey in Japan, reports of alleged adverse events were made in 2584 cases out of a total of 8.9 million HPV vaccine doses (0.03% of total doses). Of those reporting adverse events, approximately 90% have had complete recovery (186 persons are still receiving medical care related to adverse events). In short, 2 people out of 100,000 administered doses (0.002%) have reported long-term health effects.
- Safety data using a large-scale re-examination survey conducted by the European Medicine Agency (EMA) and in France revealed no difference in the occurrence rates of severe adverse reactions caused by HPV vaccines between vaccinated and unvaccinated cohorts. The same result was found in a study conducted by Nagoya City in Japan.

A mother's voice whose daughter experienced cervical cancer

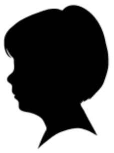

My daughter was diagnosed with cervical cancer at a medical examination at the age of 29 years. She underwent a total hysterectomy and lost her uterus. It happened about a year after she got married. She was hoping to have children but was robbed of this dream.

Fortunately, the cancer was found early and her life was saved. However, this experience continues to impact her life. Every time the phone rings, she is terrified it is her oncologist calling and she holds her breath until she gets the results. I am hopeful that she will live a long and healthy life, but thoughts of the cancer returning are always lurking in the back of our minds.

I do not want others to have the same difficult experience as my daughter. If I could, I would have had my daughter vaccinated with the HPV vaccine. I always say to my friends, "Please have your daughter receive the HPV vaccine to prevent cervical cancer."

We recommend HPV vaccination to protect your child from cervical cancer.
